# Emergence and genomic adaptation of the globally disseminated ST2250 lineage within the *Staphylococcus aureus* complex

**DOI:** 10.1128/aac.01628-25

**Published:** 2026-01-26

**Authors:** Wangxiao Zhou, Dizhong Chen, Xu Dong, Ting Yang, Caixia Liu, Ye Jin, Deru Lei

**Affiliations:** 1 Clinical Laboratory Center, The Second Affiliated Hospital & Yuying Children’s Hospital of Wenzhou Medical University 26452, Wenzhou, Zhejiang, People’s Republic of China; 2 State Key Laboratory for Diagnosis and Treatment of Infectious Diseases, National Clinical Research Center for Infectious Diseases, Collaborative Innovation Center for Diagnosis and Treatment of Infectious Diseases, The First Affiliated Hospital, Zhejiang University School of Medicine 71069, Hangzhou, People’s Republic of China; 3 Department of General Intensive Care Unit, The Second Affiliated Hospital of Zhejiang University School of Medicine 89681, Hangzhou, Zhejiang, People’s Republic of China; 4 Key Laboratory of Early Warning and Intervention of Multiple Organ Failure, China National Ministry of Education, Hangzhou, Zhejiang, People’s Republic of China; The Peter Doherty Institute for Infection and Immunity, Melbourne, Victoria, Australia

**Keywords:** *Staphylococcus argenteus*, sequence type 2250, genomic analysis, antimicrobial resistance, virulence, adaptive evolution

## Abstract

*Staphylococcus argenteus*, a member of the *S. aureus* complex, is increasingly recognized as a globally distributed pathogen with significant clinical relevance. Among its lineages, sequence type (ST) 2250 has emerged as the most prevalent and geographically widespread, yet its evolutionary history and genomic adaptations remain incompletely understood. In this study, we conducted a comprehensive genomic analysis of 277 ST2250 genomes from 26 countries between 2008 and 2025, integrating 14 newly sequenced isolates from China. Phylogenetic reconstruction resolved a basal clade I around 1989 and sister clades II and III that diversified later, in approximately 1996 and 1997, with frequent cross-regional, intercontinental, and cross-host transmission events. A methicillin-resistant *S. argenteus* subclade within clade II likely arose from a single SCC*mec* IVc acquisition, accompanied by a *blaZ*-carrying plasmid. Clade III genomes carried a related multidrug-resistant (MDR) plasmid encoding *blaZ*, *tet*(L), and *aph(3')-III*; Bayesian phylogenetic inference indicated that this plasmid was introduced into the ancestor of the clade III MDR subclade around 2001, potentially promoting its subsequent expansion. Both clades also exhibited enriched virulence profiles, particularly the secretion system gene *esaG7*. Despite the widespread presence of active defense systems that might limit the acquisition of mobile genetic elements, the ST2250 pan-genome remains open, with evidence of active gene flux and convergent selection targeting resistance, virulence, and metabolic pathways. These findings elucidate the global spread, ecological plasticity, and adaptive evolution of ST2250, providing critical genomic insights into the emergence and persistence of this lineage.

## INTRODUCTION

*Staphylococcus argenteus* is a recently delineated member of the *S. aureus* complex (SAC), which also includes *S. aureus*, *S. schweitzeri*, *S. roterodami*, and *S. singaporensis* (1, 2). Initially recognized in 2006 during molecular epidemiological surveillance of methicillin-resistant *S. aureus* (MRSA) in northern Australia, *S. argenteus* was originally classified as a clonal complex 75 of *S. aureus* due to their close phenotypic similarity (3). However, subsequent whole-genome sequencing (WGS) analyses revealed substantial genetic divergence, leading to its formal designation as a separate species in 2015 (4). Since its recognition, reports of *S. argenteus* infection have emerged across diverse regions including Oceania, Asia, Europe, Africa, and the Americas (5–8). In clinical isolates from Asia, the proportion of *S. argenteus* typically remains below 10% among presumptive *S. aureus* isolates (5, 6, 9). Nevertheless, comprehensive epidemiological data from China remain limited, and the clinical relevance of *S. argenteus* infections in this region is still not fully understood. A few recent studies have detected *S. argenteus* in clinical infections and retail food samples in southern China (10, 11), raising public health concerns regarding potential foodborne or zoonotic transmission routes.

Although *S. argenteus* was initially considered less virulent than *S. aureus*, largely due to the absence of staphyloxanthin (12), accumulating evidence now indicates that it can cause a broad spectrum of severe infections, including osteomyelitis, prosthetic joint infections, infective endocarditis, mycotic aneurysm, and bloodstream infections (13). In fact, more recent clinical data show that patients with *S. argenteus* bacteremia experience higher mortality rates than those infected with methicillin-susceptible *S. aureus* (MSSA) (14). Experimental studies have demonstrated that *S. argenteus* and *S. schweitzeri* exhibit significantly greater cytotoxicity toward human cells, which has been linked to a 12- to 15-fold increase in the expression of α-hemolysin compared with *S. aureus* (15). A comparative genomic study has shown that approximately 76% of the 111 virulence-associated genes typically found in *S. aureus* are also present in *S. argenteus* (16). Beyond virulence, *S. argenteus* can exhibit various resistance due to the presence of genes such as *blaZ*, *fusA,* and *tet*(L) (17), underscoring its growing potential as both a pathogen and a reservoir for antimicrobial resistance.

Among the currently recognized lineages of *S. argenteus*, sequence type (ST) 2250 has emerged as the most dominant and globally disseminated clone in recent years (17). In Thailand, where it has been most extensively studied, ST2250 isolates are typically susceptible to methicillin but resistant to penicillin (18). By contrast, isolates from Australia and the UK frequently harbor staphylococcal cassette chromosome *mec* (SCC*mec*) type IV, conferring methicillin resistance (19). However, these observed patterns could be influenced by biased isolate selection and may not accurately reflect regional resistance trends. Beyond resistance, ST2250 has also been implicated in persistent infections, including cases of prosthetic joint infection where isolates demonstrated small-colony variant formation and increased intracellular persistence that may be further induced under antibiotic exposure (20). Such features may facilitate chronic infection and complicate treatment. Despite its prominence, most previous research has focused on *S. argenteus* at the species level, leaving lineage-specific traits underexplored. A focused investigation of the ST2250 genome is needed to determine whether this lineage possesses unique evolutionary, resistance, or virulence characteristics that contribute to its apparent success.

To elucidate the evolutionary mechanisms and genomic diversity of *S. argenteus* ST2250, we conducted a large-scale comparative genomic analysis of 277 genomes, including newly sequenced local clinical isolates and publicly available global data. Through phylogenetic reconstruction and molecular evolutionary analyses, we delineated its population structure, geographic dissemination, and patterns of adaptive evolution. We further examined the distribution of antimicrobial resistance (AMR) genes, virulence factors, mobile genetic elements (MGEs), defense systems, and genes under positive selection to investigate the genomic basis of its clinical success and ecological adaptability. This study provides a foundational resource for understanding the evolutionary trajectory and genetic makeup of ST2250, contributing essential insights for combating *S. argenteus* infections.

## MATERIALS AND METHODS

### Collection of bacterial isolates

Between October 2024 and April 2025, a total of 435 consecutive, non-duplicate presumptive *S. aureus* complex isolates were prospectively collected from clinical specimens submitted for routine diagnostic testing at the Second Affiliated Hospital and Yuying Children’s Hospital of Wenzhou Medical University (Zhejiang Province, China). Duplicate isolates were defined as multiple isolates of the same species from the same patient during the study period, and only the first isolate per patient was retained. No additional selection criteria were applied. Initial species identification was performed using MALDI-TOF MS (Bruker, Bremen, Germany). Antimicrobial susceptibility testing (AST) was conducted using the VITEK-2 automated system (bioMérieux, France) following the manufacturer’s protocols. In addition, AST for daptomycin and ceftobiprole was performed by broth microdilution in accordance with CLSI guidelines.

### Whole-genome sequencing and genomic analysis

Whole-genome sequencing of all 435 presumptive *S. aureus* complex isolates was carried out using the Illumina HiSeq X Ten platform (Illumina, San Diego, CA, USA) with 2 × 150 bp paired-end reads. Raw sequencing data were processed using fastp v0.23.2 (21) for adapter trimming and quality filtering (minimum Phred score ≥20), followed by *de novo* assembly with SPAdes v3.14.1 (22). Species-level classification was performed using GTDB-Tk v2.4.0 (23), which identified 17 isolates as *S. argenteus*; the remaining isolates were confirmed as *S. aureus*. *In silico* multi-locus sequence typing (MLST) of *S. argenteus* was performed using the MLST 2.0 webserver (https://cge.food.dtu.dk/services/MLST/), which revealed that 14 of the 17 *S*. *argenteus* isolates belonged to the ST2250 lineage. These ST2250 isolates were selected for downstream comparative analyses. Additionally, long-read sequencing of a representative ST2250 isolate (strain FEYWZ357) was conducted on the Oxford Nanopore MinION platform, and hybrid assembly of the complete genome was achieved by integrating Illumina short-read and Nanopore long-read data using Unicycler v0.5.0 (24).

To expand the data set, 450 publicly available *S. argenteus* genomes were downloaded from the NCBI genome database (as of July 2024). *In silico* MLST was conducted on these genomes, and only ST2250 genomes were retained for further phylogenomic analysis, resulting in a final data set of 277 ST2250 genomes (Table S1).

*In silico* typing of SCC*mec* elements and *spa* types was carried out using SCCion (https://github.com/esteinig/sccion). Genome annotation was performed using Bakta v1.11.2 (25), and pan-genome analysis of ST2250 genomes was conducted in strict mode using Panaroo v1.3.0 (26). The evolutionary dynamics of gene exchange within the ST2250 lineage were assessed using Panstripe v0.3.0 (27), applying a generalized linear model with a Tweedie distribution. The bacterial defense and anti-defense systems were examined across all ST2250 genomes using DefenseFinder v2.0.1 (https://github.com/mdmparis/defense-finder).

### Phylogenetic analysis of ST2250 lineage

Alignment of 277 ST2250 genomes against the reference genome *S. argenteus* TWCC 58113 (GenBank accession no. AP018562.1) was performed using Snippy v4.6.0 (https://github.com/tseemann/snippy) to extract core-genome SNPs. Gubbins v2.4.1 (28) was used to identify regions undergoing recombination within the core genome, and the resulting recombination-free core-genome SNPs were employed to construct the phylogenetic tree. Maximum-likelihood phylogenies were inferred using RAxML v8.2.12 (29) under the GTRGAMMAIX model with 1,000 bootstrap replicates. The genome of *S. argenteus* MSHR1132 (ST1850, GenBank: FR821777.2) was used as the outgroup. The dated phylogeny based on Bayesian inference was constructed using BactDating v1.1 (30) under a mixed model with 100 million cycles. Convergence of the model was confirmed by ensuring effective sample sizes >200 for all parameters. Geographic origin was inferred using the Bayesian Binary MCMC method implemented in RASP v4.2 (31), employing 10 parallel chains, each run for 50 million generations. A minimum spanning tree (MST) of the 277 genomes was constructed using PHYLOViZ v2.0 (https://online.phyloviz.net/index) to visualize pairwise genetic relationships.

### Comparison of AMR genes, virulence genes, and MGEs among ST2250 lineage

AMR genes and virulence factors were identified using ABRicate v1.0.0 (https://github.com/tseemann/abricate) based on ResFinder and VFDB databases with default settings. Putative pathogenicity islands and prophage elements were identified using BLASTN (https://blast.ncbi.nlm.nih.gov/Blast.cgi), with published *S. aureus* MGE sequences as references and thresholds set at ≥85% nucleotide identity and query coverage.

In addition, all available *S. aureus* genome sequences (*n* = 85,893 as of July 2024) were downloaded from the NCBI GenBank database. STs were assigned and genomes were annotated as described above for *S. argenteus*. We used Assembly Dereplicator v0.3.1 (Mash distance threshold 0.001; https://github.com/rrwick/Assembly-Dereplicator) to cluster and dereplicate global ST59, ST8, and ST239 genomes, selecting 206 ST59, 241 ST8, and 136 ST239 representatives for comparison with ST2250. Methods for identifying AMR genes, virulence genes, and MGEs were the same as those applied to ST2250.

### Positive selection analysis

Ancestral state reconstruction of all core genome SNPs within ST2250 lineage was performed using PAML 4 (32), and each SNP was subsequently categorized as intergenic, synonymous, or nonsynonymous based on its annotation and inferred ancestral state. Nonsynonymous to synonymous substitution (dN/dS) ratios were calculated using the NY98 model, which incorporates both codon usage and transition/transversion rate biases. Genes were considered to be under positive selection if the adjusted dN/dS ratio exceeded 1.5, or if no synonymous mutations were detected but at least seven nonsynonymous mutations were present.

### Genetic origin analysis

For origin analysis of clonal complex (CC) 2250 (denoting ST2250 and its single locus variants), we computed Mash distances v2.3 (33) between TWCC 58113 and all available *S. aureus* (*n* = 85,893) and non-ST2250 *S. argenteus* (*n* = 173) genomes, selected the 500 closest genomes together with 277 ST2250 genomes for downstream phylogeny, and inferred recombination events with Gubbins v2.4.1 (28) as described above for ST2250.

### Statistical analysis

Statistical analysis was conducted using the Wilcoxon rank-sum test for continuous variables and the chi-square or Fisher’s exact test for categorical variables, with *P* values <0.05 considered statistically significant. All data were processed and analyzed using R software (version 4.5.1).

## RESULTS

### Identification and prevalence of *S. argenteus* isolates

Between October 2024 and April 2025, a total of 435 consecutive, non-duplicate *S. aureus* complex isolates were obtained from a large tertiary teaching hospital in Zhejiang Province, China. Species identification by MALDI-TOF MS followed by WGS revealed that 17 isolates (3.91%) were *S. argenteus*. These isolates were recovered from various clinical specimens, including blood (*n* = 8), respiratory tract samples (*n* = 5), wound/pus exudates (*n* = 2), and one specimen each from the urinary tract and bile. All patients received antimicrobial therapy, but five (29.41%) died during hospitalization from *S. argenteus* infection, including three with sepsis and two with pneumonia. The clinical information of these patients is provided in Table 1.

**TABLE 1:** Clinical information of 17 patients carrying *S. argenteus* isolates

Isolate	Isolation date	ST	SCC*mec* type	*spa* type	Age (year)	Gender	Specimen	Ward	Primary disease	Outcome
FEYWZ100	2024-11-27	ST2250_1LV^*a*^	MSSArg	t5078	81	Male	Respiratory tract	Critical care medicine	Pneumonia	Discharged
FEYWZ123	2024-12-06	ST2250	MSSArg	t7960	16	Male	Respiratory tract	Emergency medicine	Pneumonia	Discharged
FEYWZ132	2024-12-11	ST2250	MSSArg	t6675	56	Female	Respiratory tract	Integrated rehabilitation	Pneumonia	Discharged
FEYWZ175	2024-12-30	ST2250	MSSArg	Undetermined	29	Male	Respiratory tract	Emergency medicine	Cerebral hemorrhage	Discharged
FEYWZ217	2025-01-16	ST2250	MSSArg	t5078	53	Male	Wound/pus	Hand surgery	Osteomyelitis	Discharged
FEYWZ357	2025-03-10	ST2250	MSSArg	Undetermined	89	Male	Blood	Emergency medicine	Pneumonia	Dead
FEYWZ35	2024-11-04	ST2250	MSSArg	t5078	64	Female	Urine	Urology surgery	Urinary tract infection	Discharged
FEYWZ369	2025-03-19	ST2250	MSSArg	Undetermined	34	Male	Wound/pus	Colorectal and anal surgery	Perihepatic abscess	Discharged
FEYWZ372	2025-03-21	ST2250	MSSArg	t5078	55	Male	Others	Hepato-pancreato-biliary surgery	Chronic cholecystitis	Discharged
FEYWZ6	2024-10-25	ST2854	MSSArg	Undetermined	80	Male	Blood	Neurosurgery	Cerebral hemorrhage	Discharged
FEYWZ12	2024-10-20	ST2250	MSSArg	t5078	83	Male	Blood	Critical care medicine	Pneumonia	Dead
FEYWZ14	2024-10-23	ST2250	MSSArg	t6675	76	Female	Respiratory tract	Neurology	Cerebral infarction	Discharged
FEYWZ15	2024-10-28	ST2250	MSSArg	t5078	53	Female	Blood	Hematology	Sepsis	Dead
FEYWZ17	2024-10-29	ST2250	MSSArg	t5078	74	Male	Blood	Infectious diseases	Sepsis	Dead
FEYWZ55	2024-11-12	ST2250	MSSArg	t7960	60	Male	Blood	Nephrology	Obstructive nephropathy	Discharged
FEYWZ65	2024-11-15	ST1223	IVb (2B)	Undetermined	41	Female	Blood	Emergency medicine	Sepsis	Dead
FEYWZ82	2024-11-21	ST2250	MSSArg	t5078	49	Male	Blood	Emergency medicine	Sepsis	Discharged

^*a*^ ST2250_1LV: one-locus variants of ST2250.

*In silico* MLST revealed that the majority of the *S. argenteus* isolates belonged to ST2250 (82.35%, 14/17), while the remaining three isolates were classified as ST1223, ST2854, and a single-locus variant of ST2250. Among the 14 ST2250 isolates, the most common *spa* type was t5078 (50%, 7/14), followed by t6675 (*n* = 2), t7960 (*n* = 2), and three unassigned strains. Notably, pairwise SNP distances among ST2250 isolates ranged from 108 to 290, well above the ≤24 SNP threshold commonly used to infer recent clonal transmission in *S. aureus* (34), indicating no evidence of recent direct transmission in our cohort and supporting independent introductions. AST showed that all *S. argenteus* isolates were susceptible to vancomycin, linezolid, tigecycline, quinupristin-dalfopristin, fluoroquinolones, rifampin, trimethoprim-sulfamethoxazole, daptomycin, and ceftobiprole. However, resistance was observed in 64.71%, 47.06%, and 35.29% of isolates to penicillin, tetracycline, and amikacin, respectively. One methicillin-resistant isolate (FEYWZ65), assigned to ST1223, was also identified (Table 2). Given the predominance of the ST2250 lineage in both this study and global epidemiology (35), ST2250 isolates were selected for subsequent genomic analyses.

**TABLE 2:** Antimicrobial susceptibility of 17 ST2250 isolates from our collection^*a*^

Isolate	OXA	PEN	ERY	CLI	RIF	TCY	TGC	CIP	LVX	MFX	GEN	AMK	SXT	LNZ	Q/D	VAN	DAP	BPR
FEYWZ100	S	R	S	S	S	R	S	S	S	S	S	S	S	S	S	S	S	S
FEYWZ123	S	R	R	R	S	R	S	S	S	S	S	R	S	S	S	S	S	S
FEYWZ132	S	R	S	S	S	S	S	S	S	S	S	S	S	S	S	S	S	S
FEYWZ175	S	S	S	S	S	S	S	S	S	S	S	S	S	S	S	S	S	S
FEYWZ217	S	S	S	S	S	S	S	S	S	S	S	S	S	S	S	S	S	S
FEYWZ357	S	R	S	S	S	R	S	S	S	S	S	R	S	S	S	S	S	S
FEYWZ35	S	R	S	S	S	S	S	S	S	S	S	R	S	S	S	S	S	S
FEYWZ369	S	R	S	S	S	R	S	S	S	S	S	S	S	S	S	S	S	S
FEYWZ372	S	S	S	S	S	S	S	S	S	S	S	S	S	S	S	S	S	S
FEYWZ6	S	S	S	S	S	S	S	S	S	S	S	S	S	S	S	S	S	S
FEYWZ12	S	R	S	S	S	R	S	S	S	S	S	R	S	S	S	S	S	S
FEYWZ14	S	S	S	S	S	S	S	S	S	S	S	S	S	S	S	S	S	S
FEYWZ15	S	R	S	S	S	R	S	S	S	S	S	S	S	S	S	S	S	S
FEYWZ17	S	R	S	S	S	S	S	S	S	S	S	R	S	S	S	S	S	S
FEYWZ55	S	S	S	S	S	S	S	S	S	S	S	S	S	S	S	S	S	S
FEYWZ65	R	R	S	S	S	R	S	S	S	S	S	S	S	S	S	S	S	S
FEYWZ82	S	R	S	S	S	R	S	S	S	S	S	R	S	S	S	S	S	S

^*a*^ R, resistant; S, susceptible; OXA, oxacillin; PEN, penicillin G; ERY, erythromycin; CLI, clindamycin; RIF, rifampin; TCY, tetracycline; TGC, tigecycline; CIP, ciprofloxacin; LVX, levofloxacin; MFX, moxifloxacin; GEN, gentamicin; AMK, amikacin; SXT, trimethoprim-sulfamethoxazole; LNZ, linezolid; Q/D, quinupristin-dalfopristin; VAN, vancomycin; DAP, daptomycin; BPR, ceftobiprole.

### Geographical dissemination and phylogenomic diversity of the ST2250 lineage

**Fig 1:**
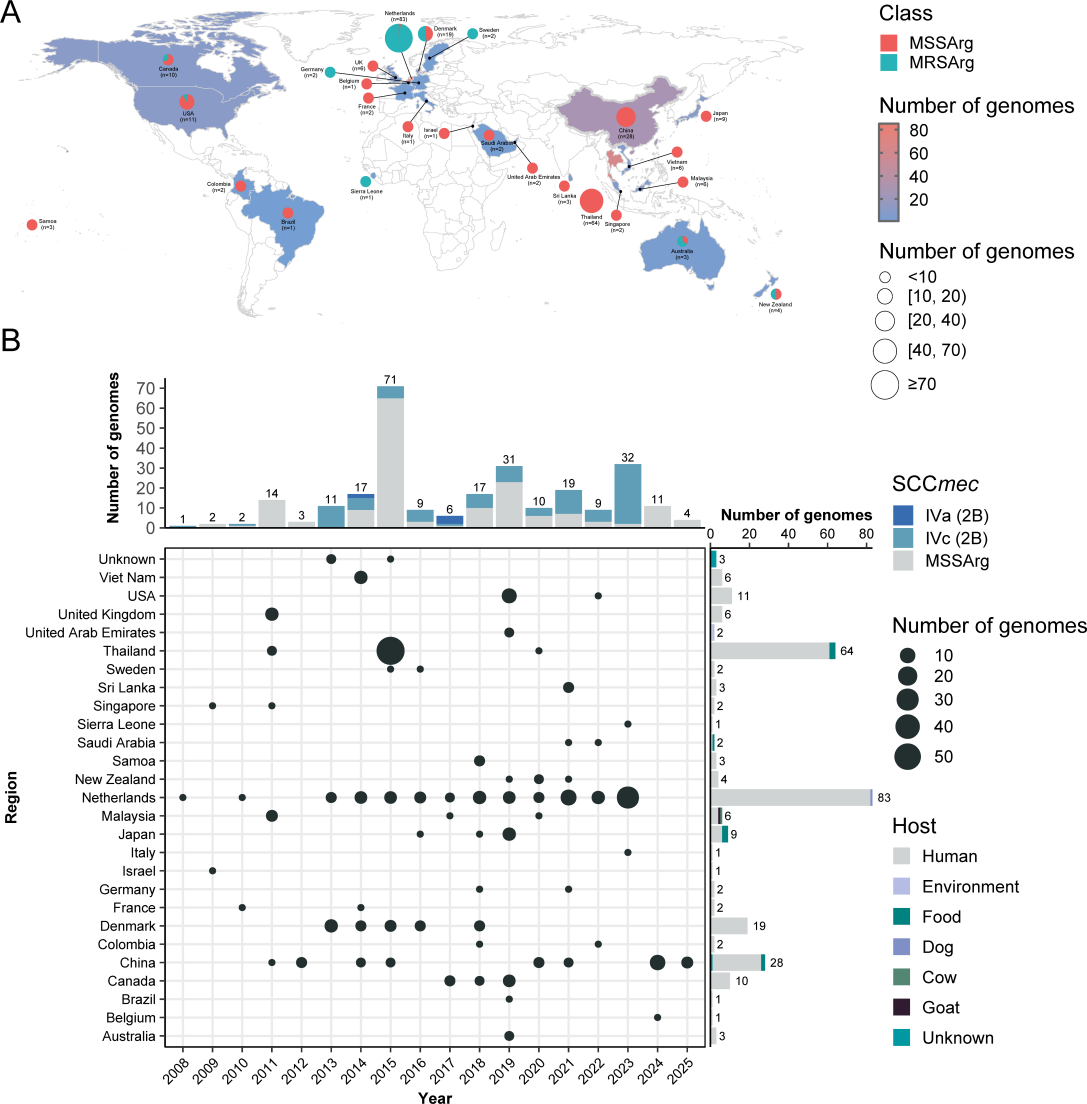
Global distribution and temporal dynamics of *S. argenteus* ST2250 genomes. (**A**) Geographic location of 277 *S*. *argenteus* ST2250 genomes collected from 26 countries. Red and cyan circles represent methicillin-susceptible (MSSArg) and methicillin-resistant (MRSArg), respectively. Circle size corresponds to the number of genomes from each country, and background shading indicates regions with ST2250 detections. (**B**) Temporal and geographic overview of ST2250 genomes. The central bubble plot displays the number of genomes (bubble size) reported per country each year. The upper bar chart shows the annual counts of genomes by SCC*mec* type. The right-side bar chart summarizes the host distribution for each country.

To investigate the global prevalence of ST2250 lineage, all publicly available genomes labeled *S. argenteus* were retrieved from the NCBI GenBank database. Among 450 genomes screened, 263 were identified as ST2250 based on *in silico* MLST analysis. Combined with 14 ST2250 isolates from our collection, a data set comprising 277 high-quality ST2250 genomes was constructed for downstream analyses. These genomes were sampled between 2008 and 2025 from 26 countries across six continents (Fig. 1), with the highest numbers reported from the Netherlands (29.96%, 83/277), Thailand (23.10%, 64/277), and China (10.11%, 28/277). Notably, 106 of the 277 genomes (38.27%) were identified as methicillin-resistant *S. argenteus* (MRSArg), originating from 10 countries across Europe, North America, and Oceania. MRSArg genomes were detected nearly every year since 2008 (Fig. 1), suggesting a sustained pattern of geographic diversity and dissemination. In addition, although most genomes were sampled from human clinical samples, 13 originated from non-human sources, including food (*n* = 9), hospital environment (*n* = 1), cow (*n* = 1), dog (*n* = 1), and goat (*n* = 1); the source of four genomes was not reported.

**Fig 2:**
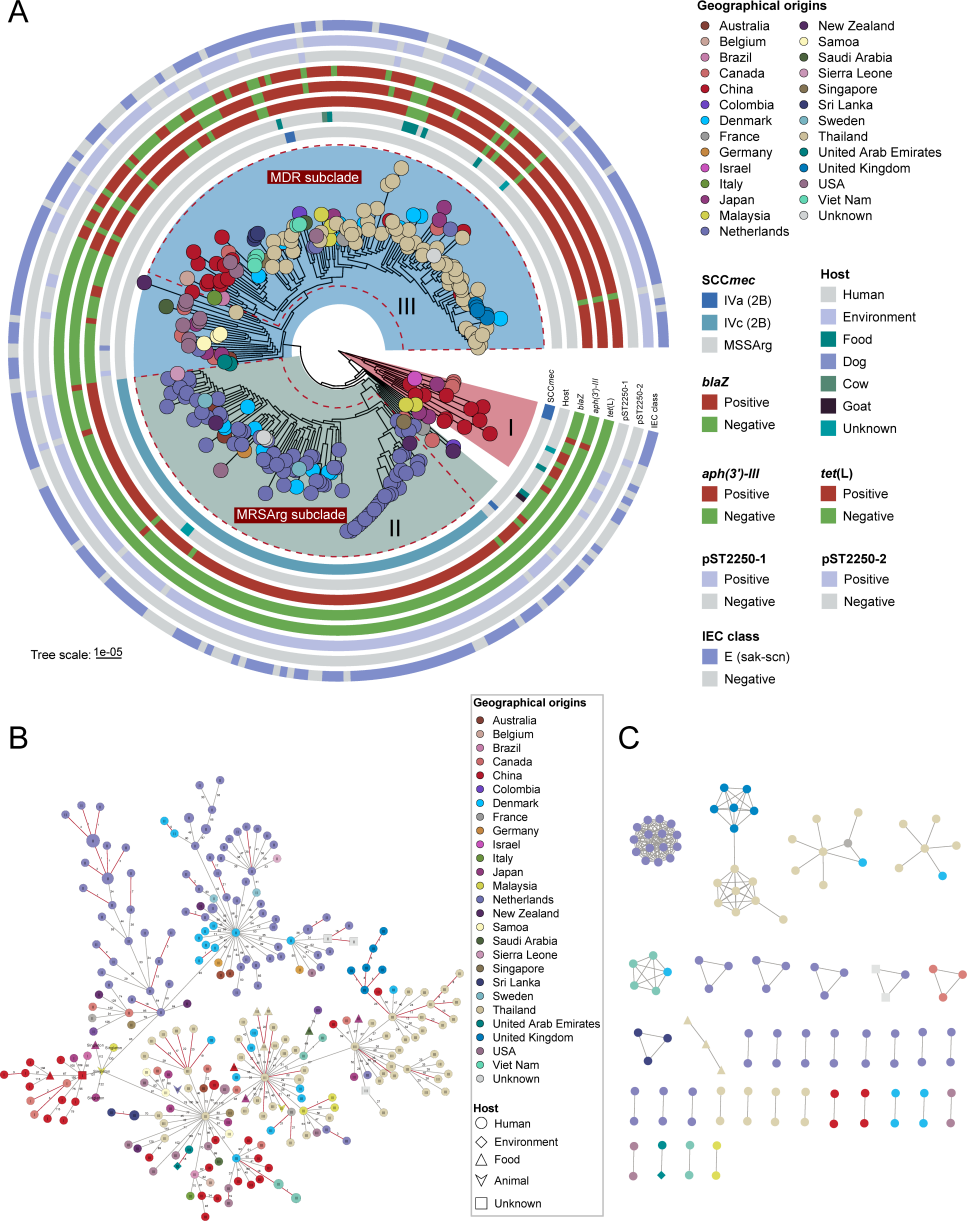
Phylogenetic and transmission analyses of 277 *S*. *argenteus* ST2250 genomes. (**A**) Phylogenetic structure of ST2250 genomes. Clade I is basal, and clades II and III share a recent common ancestor. Tip colors represent the country of isolation, and the concentric rings (inner to outer) indicate SCC*mec* types, host types, presence/absence of resistance genes [*blaZ*, *aph(3′)-III* and *tet*(L)], plasmids (pST2250-1 and pST2250-2), and immune evasion cluster (IEC) gene classes. MRSArg and MDR subclades are highlighted. (**B**) MST of all ST2250 genomes. Each node represents one genome, with node color denoting geographic origin and shape indicating host source. Red edges denote potential transmission clusters, defined by a <24 SNP threshold. (**C**) Transmission clusters based on pairwise SNP distances <24. Each node represents one genome, colored by geographic origin and shaped by host source.

Maximum-likelihood phylogenetic analysis based on core-genome SNPs resolved three clades (I to III; Fig. 2A) within the 277 ST2250 genomes. The time to the most recent common ancestor was estimated at 1986 (95% highest posterior density [HPD]: 1981–1990; Fig. S1). Clade I branched near the root, emerging shortly after the ST2250 ancestor around 1989, whereas clades II and III formed a sister group that diversified slightly later in the mid to late 1990s, at approximately 1996 and 1997, respectively. These findings indicate that the global spread of ST2250 has persisted for more than three decades. After excluding recombinant regions, the estimated mean nucleotide substitution rate for the ST2250 lineage was 1.58 × 10^−6^ substitutions/site/year (95% HPD: 1.41 × 10^−6^ to 1.75 × 10^−6^), comparable to that of other prevalent *S. aureus* lineages (36). Phylogeographic analysis indicated that clade I genomes originated from only four countries, predominantly China (68.75%, 11/16), whereas clades II and III exhibited greater geographic diversity. Notably, clade II was dominated by genomes from the Netherlands (73.83%, 79/107), while clade III included a high proportion of genomes from Thailand (42.67%, 64/150) and China (11.33%, 17/150). Additionally, aside from sporadic MRSArg carrying SCC*mec* IVa (*n* = 6), all MRSArg harboring SCC*mec* IVc were clustered in clade II, forming a distinct MRSArg subclade, likely resulting from a single acquisition event of SCC*mec* IVc around 2001.

In addition, the paired SNP differences of genomes between three clades varied from 96 to 321 (median = 160), indicating considerable genetic divergence at the clade level. However, genomes originating from geographically distant regions, including multiple countries and continents, were tightly clustered within clades II and III (Fig. 2A and B), suggesting potential cross-country transmission. Phylogenetic analysis also showed that non-human genomes were scattered across the phylogeny and clustered with proximal human-derived genomes, indicating potential cross-host transmission. Based on a ≤24 SNP threshold, we identified 36 independent transmission events across 12 countries (Fig. 2C), including intercontinental links between Thailand and the United Kingdom, Thailand and Denmark, and Vietnam and Denmark, as well as a potential transmission event involving a patient and the clinical environment in the United Arab Emirates (37). Notably, Bayesian phylogeographic analysis supports a Japanese origin for clade I Chinese genomes and implicates a basal Singapore methicillin-susceptible *S. argenteus* (MSSArg) as a potential ancestor of the Dutch ST2250 MRSArg expansion, subject to sampling bias (Fig. S2).

To probe the origin of CC2250 (denoting ST2250 and its single locus variants), we reconstructed a phylogeny including the 500 closest hits to the ST2250 reference TWCC 58113 together with 277 ST2250 genomes (Fig. S3A). Within CC2250, the median pairwise core SNP distance was 131 (range 0 to 312). The closest external lineage was CC75, with a median distance of 11,126 SNPs (range 11,071 to 11,208) from CC2250 (Fig. S3B), far exceeding within CC diversity. Recombination mapping along the TWCC 58113 reference showed no single mosaic block indicative of wholesale chromosomal replacement between CC2250 and CC75; inferred recombination events were dispersed across the chromosome (Fig. S3C).

### Clade-specific distribution of resistance genes among ST2250 lineage

**Fig 3:**
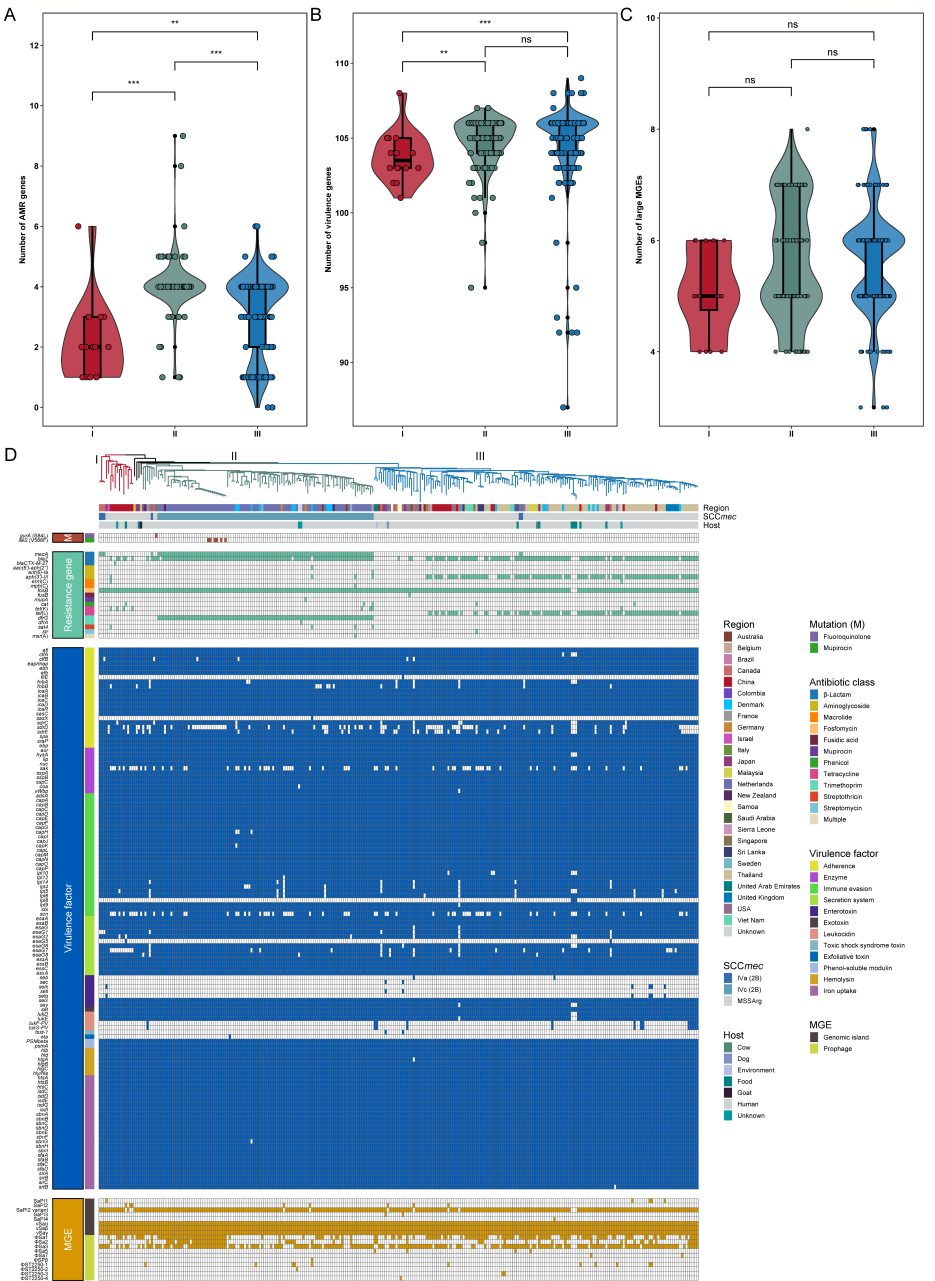
Distribution of AMR genes, virulence factors, and large MGEs in ST2250 genomes. (**A–C**) Violin plots comparing the number of AMR genes (**A**), virulence factors (**B**), and large MGEs (**C**) among the three ST2250 clades. **, *P* < 0.01; ***, *P* < 0.001. ns, not significant. (**D**) Heatmap illustrating the distribution of mutations, AMR genes, virulence factors, and MGEs across ST2250 lineages. Squares colored by trait category indicate the presence of each examined trait.

Given the critical role of multidrug resistance in the persistence of *S. argenteus* in healthcare settings, we compared the resistance gene profiles across major clades within the ST2250 lineage. A total of 22 resistance genes were identified, covering 12 classes of antimicrobials. Notably, the mean number of resistance genes per genome was higher in clades II (4.05) and III (3.12) than in clade I (2.12) (*P* < 0.001) (Fig. 3A). Specifically, the high prevalence of MRSArg in clade II (94.39%) was associated with frequent carriage of the β-lactam resistance gene *mecA* (94.39%) and the trimethoprim resistance gene *dfrG* (93.46%), both located within the SCC*mec* IVc element; and the *blaZ* gene encoding β-lactamase was detected in 94.39% of clade II genomes, significantly higher than in clade I. In addition, clade III genomes showed a high prevalence of *blaZ* (74.67%), the tetracycline resistance gene *tet*(L) (63.33%), and the aminoglycoside resistance gene *aph(3')-III* (70.67%), which were enriched within a distinct multidrug-resistant (MDR) subclade (Fig. 2A and 3D).

Further analysis revealed that *blaZ* in clade II was located on a multi-replicon plasmid (rep5a/rep16), designated pST2250-1. This plasmid, approximately 20.6 kb in size, also harbored the heavy metal resistance genes *cadD* and *arsR* (Fig. S4A). BLAST search results indicated that pST2250-1 shared 100% nucleotide identity with *S. aureus* plasmid pMW2 (AP004832.1), which was originally identified in a human clinical isolate representing the community-associated MRSA lineage USA400 (Fig. S4C). Interestingly, clade III genomes carried *blaZ*, *tet*(L), and *aph(3')-III* on a similar multi-replicon plasmid (rep5a/rep16), designated pST2250-2, which is approximately 30.3 kb in size (Fig. S4B). Comparative genomics indicated that pST2250-2 shared a conserved backbone with pST2250-1 but contained an additional accessory module carrying *tet*(L), *aph(3')-III*, and the heavy metal resistance gene *czcD* (Fig. S4C), suggesting a history of plasmid recombination. Moreover, pST2250-2 showed the highest sequence similarity (87% coverage and 99.53% identity) to an unnamed plasmid from *S. aureus* strain Sau41 (CP141473.1), with key differences localized to the resistance gene region, where Sau41’s plasmid carried *tet*(K) instead of *tet*(L) (Fig. S4C). Bayesian phylogenetic inference indicated that pST2250-2 was introduced into the ancestor of the MDR subclade of clade III around the year 2001 (Fig. S1) and subsequently underwent multiple gene loss events.

Aside from the fosfomycin resistance gene *fosB* (98.92%), all other resistance genes were found at low frequencies (<10%) and showed scattered presence across the ST2250 clades.

### Comparison of virulence genes in ST2250 clades

The virulence-associated gene content of each clade was characterized across all 277 ST2250 genomes to determine their pathogenic potential. A total of 119 virulence genes were identified, of which 99 (83.19%) were present in more than 90% of genomes across all clades, suggesting that these genes constitute the core virulence gene set of the ST2250 population. As shown in Fig. 3B, the number of virulence genes per genome was significantly higher in clades II (mean = 104.67) and III (mean = 104.61) than in clade I (mean = 103.75), primarily due to the elevated prevalence of the secretion system gene *esaG7* in clades II (96.26%) and III (90.67%) compared with clade I (31.25%). In addition, immune evasion cluster (IEC) genes such as *scn* and *sak* were found across all clades, with slightly higher detection rates in clades II (75.70%) and III (78.00%) than in clade I (56.25%). Notably, the PVL genes *lukF/PV* and *lukS/PV* were detected in 13 ST2250 genomes, while the exfoliative toxin gene *eta* was identified in one genome (Fig. 3D).

### Characterization of the large MGEs in ST2250 lineage

**Fig 4:**
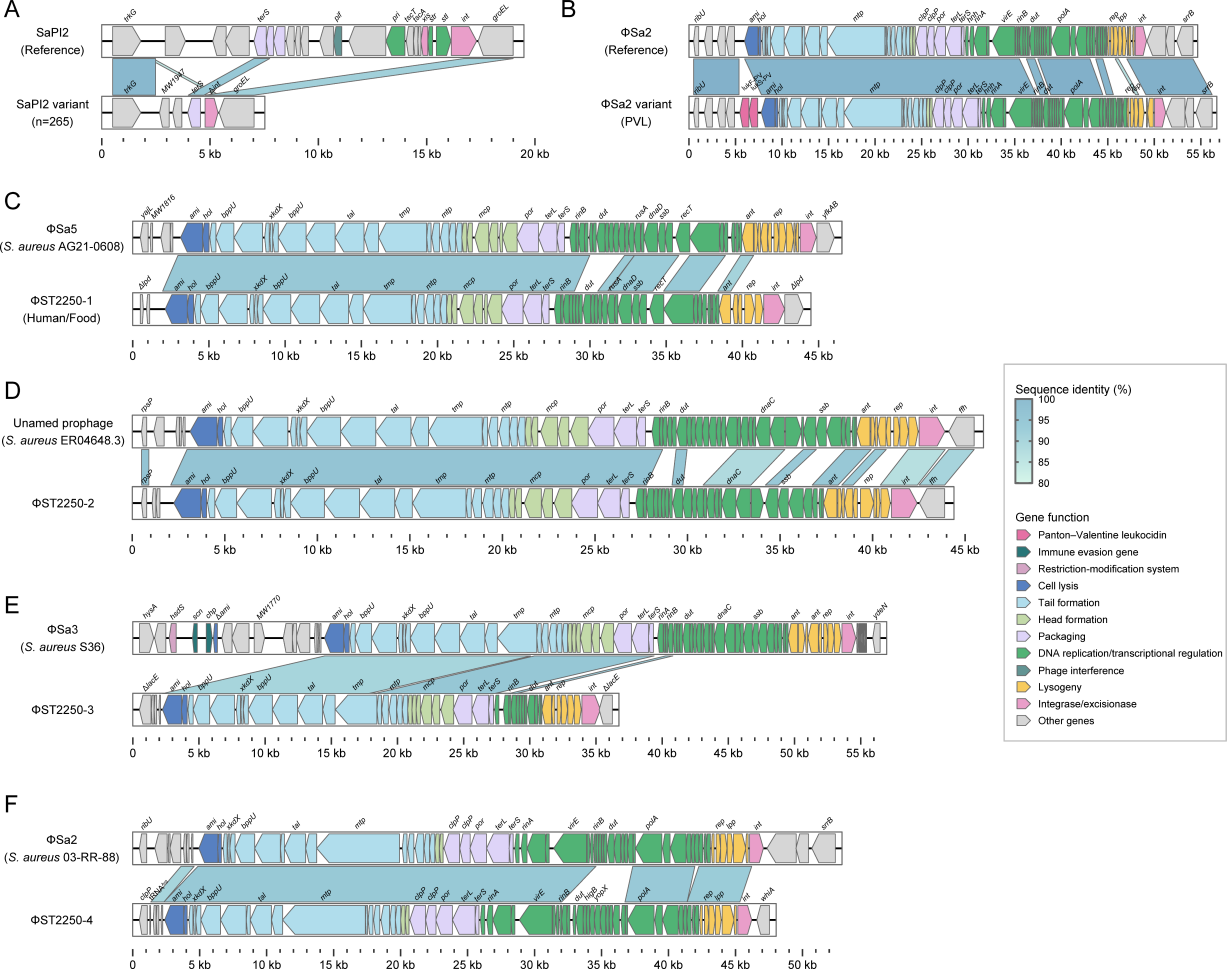
Comparative genomic analysis of SaPI and prophages in ST2250 genomes. (**A, B**) Linear comparison of SaPI2 and φSa2 regions between ST2250 genomes and their respective references. (**C–F**) Structural comparisons of four prophage elements (φST2250-1 to φST2250-4) with their closest known counterparts. Arrowed boxes represent predicted open reading frames, shaded according to functional categories.

To investigate the potential contribution of large MGEs to genomic plasticity in the ST2250 lineage, we examined the distribution of staphylococcal pathogenicity islands (SaPIs), genomic islands, and prophages across the data set. The overall number of MGEs did not differ significantly among the three clades (*P* > 0.05, Fig. 3C). As shown in Fig. 3D, the genomic islands vSaα, vSaβ, and vSaγ were found in all genomes analyzed. In contrast, the prevalence of the prophage φSa3 was relatively low across clades I to III (56.25%, 75.70%, and 77.33%, respectively). Notably, although nearly all ST2250 genomes (98.56%) carried SaPI2, only 4.33% (12/277) retained the intact structure; in over 95% of genomes, the conserved region responsible for regulation and replication was missing (Fig. 4A). In addition, one clade II genome and 12 clade III genomes were found to carry φSa2 encoding PVL (Fig. 4B). Of particular interest, one clade II genome (H1864) harbored φSPβ encoding the adhesion gene *sasX*, which was previously associated with the successful spread of hospital-associated MRSA ST239 in Asia (38).

We also identified four previously uncharacterized prophages in the ST2250 lineage, designated φST2250-1 through φST2250-4, none of which carried known resistance or virulence genes (Fig. 3D and 4C through F). Structural analysis revealed that all four prophages exhibited a highly organized genomic architecture, including regions associated with lysogeny, DNA replication and transcriptional regulation, packaging, head and tail proteins, and host cell lysis. Notably, sequence differences between φST2250-1 to φST2250-4 and their closest homologs were primarily concentrated in the DNA replication/transcriptional regulation and lysogeny modules, suggesting that these prophages may have undergone recombination during their evolutionary history.

### Distributions of defense and anti-defense systems among ST2250 clades

**Fig 5:**
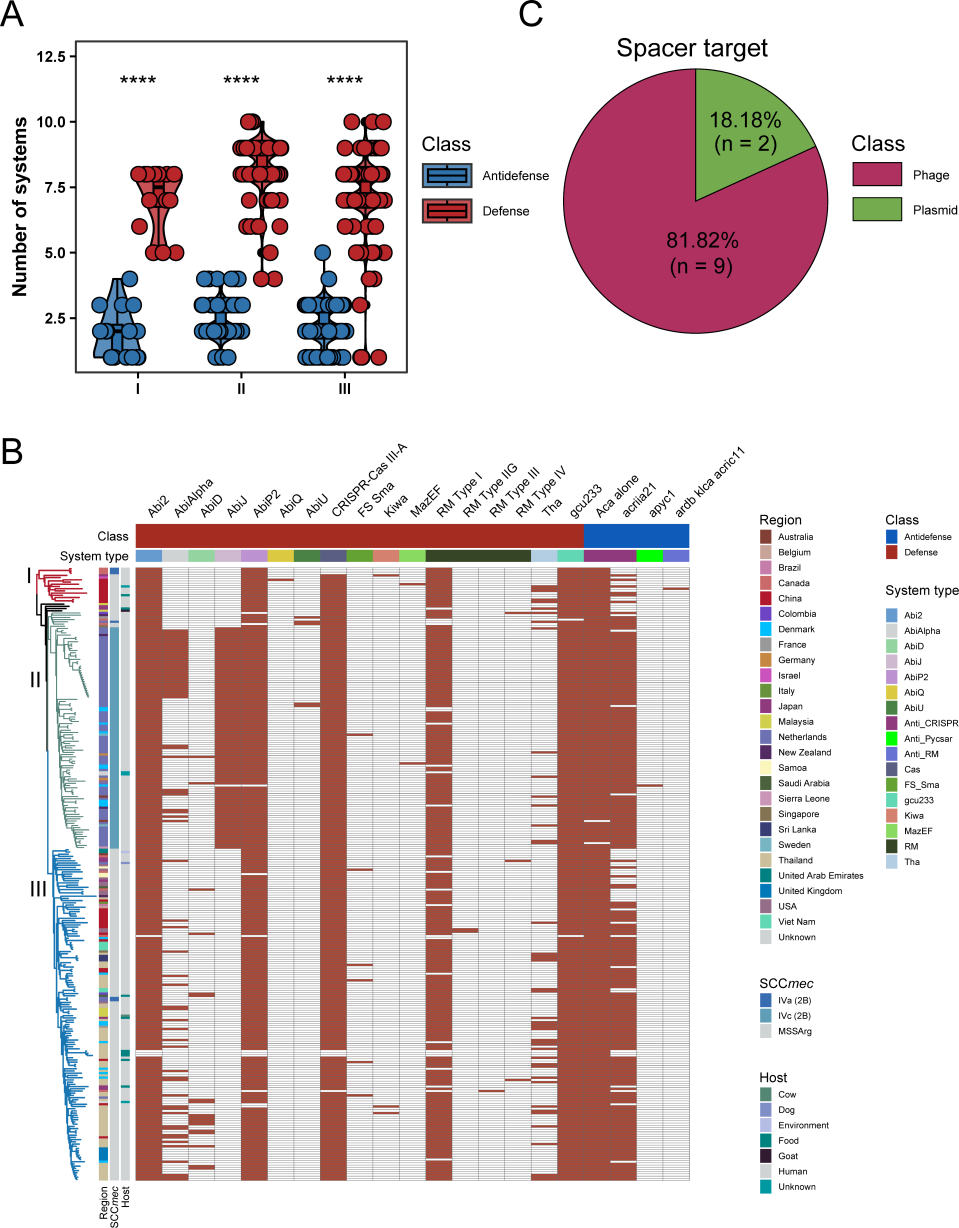
Distributions of defense and anti-defense systems among ST2250 genomes. (**A**) Quantification of defense and anti-defense systems across the three clades of ST2250. ****, *P* < 0.0001. (**B**) Heatmap illustrating the presence or absence of specific defense and anti-defense systems across all ST2250 genomes. (**C**) Pie chart summarizing the predicted targets of CRISPR spacers among ST2250 genomes.

By investigating the defense systems that potentially limit horizontal gene transfer (HGT) and the co-evolved anti-defense mechanisms in the ST2250 lineage, we identified a total of 17 defense systems and 4 anti-defense systems across the data set. In all clades, the number of defense systems per genome was significantly higher than that of anti-defense systems (*P* < 0.0001, Fig. 5A). Notably, the systems Abi2, AbiP2, type III-A CRISPR-Cas, type I RM, and gcu233 were highly prevalent (>80%) across all clades (Fig. 5B). Given that the type III-A CRISPR-Cas system in both *S. epidermidis* and *S. aureus* has been shown to protect against phage infection and plasmid transfer, we further analyzed CRISPR spacers in 270 ST2250 genomes that carried this system. We identified 11 unique spacer sequences, 9 of which shared homology with known phage sequences, and 2 of which targeted *S. aureus* plasmids (Fig. 5C; Table S2). With the exception of one spacer (IIIASP-11), all other spacers were present in >55% of CRISPR-positive genomes.

### Open pan-genome architecture of the ST2250 lineage

**Fig 6:**
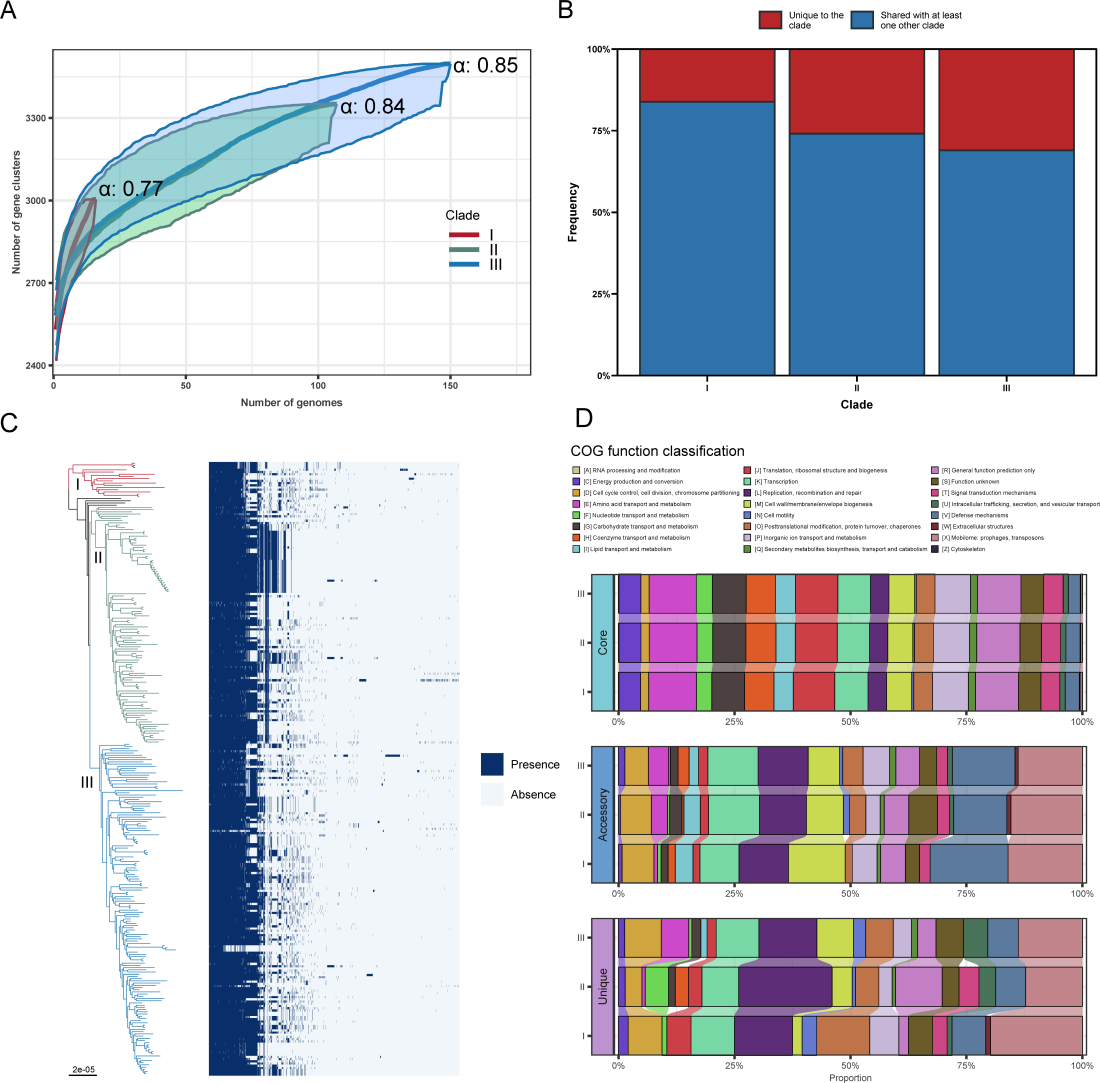
Pan-genome diversity of ST2250 genomes. (**A**) Pan-genome accumulation curves for the three clades of ST2250. The total number of gene clusters increases with the number of genomes sampled, and fitted Heaps’ law alpha values are shown for each clade. (**B**) Proportional distribution of clade-specific vs shared accessory genes across the three clades. Red bars represent genes unique to one clade, while blue bars indicate genes shared with at least one other clade. (**C**) Binary presence-absence heatmap of accessory genes across all 277 ST2250 genomes, sorted according to the core genome phylogeny. (**D**) COG functional classification of core, accessory, and unique genes for each of the three clades.

To investigate the genomic diversity of the ST2250 lineage, we constructed a comprehensive pan-genome comprising core, accessory, and unique genes across all genomes. The resulting ST2250 pangenome consisted of 3,961 genes, with core, accessory, and unique genes accounting for 54.43%, 27.34%, and 18.23%, respectively. A generalized linear model based on the time-calibrated core-genome phylogeny revealed a significant association between core-genome branch length and the number of gene exchange events (*P* = 2.98e-28), indicating that the ST2250 pan-genome is actively evolving and represents an open genomic structure. Furthermore, pan-genome accumulation curves for each clade continued to rise without reaching a plateau as more genomes were incorporated (Fig. 6A), reinforcing the open nature of the ST2250 pan-genome. This interpretation was further supported by α-values derived from Heap’s law fitting, all of which were below 1 (*α* = 0.77, 0.84, and 0.85 for clades I, II, and III, respectively). After excluding clade-specific core genes, we found that 16.16% to 31.02% of the variable genes were clade-specific (Fig. 6B and C), indicating moderate interclade variability in the pan-genome.

To assess the functional composition of the pan-genome, we performed COG annotation for each clade (Fig. 6D). The functional profiles of core genes were highly similar across clades; in contrast, the accessory and unique genes displayed greater functional diversity across clades. Compared to the core genome, the accessory and unique gene pools of different clades contained a larger number of genes related to functional categories such as “Mobilome: prophages, transposons” and “Defense mechanisms.”

### Positive selection analysis of the ST2250 lineage

To further elucidate the evolutionary trajectory of the ST2250 population, we investigated signatures of convergent evolution that may reflect shared adaptive responses to selective pressures. A total of 39 genes were predicted to be under positive selection, with adjusted dN/dS ratios ranging from 1.65 to 2.69 (Table S3; Fig. S5). Among these, two genes were associated with antimicrobial resistance, including *fmtB* (related to β-lactam resistance) and *tcaB* (involved in teicoplanin resistance), and two genes were linked to bacterial virulence, including *geh* (a lipase gene) and *kdpD* (encoding a sensor histidine kinase implicated in virulence regulation). The remaining 35 genes under positive selection were primarily involved in metabolic processes, particularly in pathways related to amino acid transport and metabolism, carbohydrate transport and metabolism, and energy production and conversion.

### Comparison with epidemic lineages of *S. aureus*

To provide an initial comparison with epidemic lineages of *S. aureus*, we compared ST2250 with community-associated *S. aureus* ST59 (prevalent in Asia) and ST8 (prevalent in the United States), and hospital-associated *S. aureus* ST239 (prevalent in Asia) for AMR genes, virulence genes, and large MGEs (Fig. S6). As shown in Fig. S6A, ST2250 carried a similar number of AMR genes to ST59 (*P* > 0.05) but significantly fewer than ST8 and ST239 (mean 3.39 vs 5.83 and 12.90; *P* < 0.001). The higher burden in ST8 and especially ST239 reflected a broader repertoire and higher prevalence of aminoglycoside, macrolide, and tetracycline resistance genes, together with lineage-typical mutations affecting fluoroquinolones, rifampicin, fusidic acid, and trimethoprim, whereas ST2250 uniquely carried the tetracycline resistance gene *tet*(L). Among the 160 virulence loci identified in the data set, 88 (55%) were present at >90% prevalence in all lineages; nonetheless, ST2250 harbored fewer virulence genes than the *S. aureus* lineages (Fig. S6B), notably lacking loci across multiple functional categories, including the adhesin gene *sasG*, the serine protease *spl* cluster, and a series of enterotoxin genes. Furthermore, the large MGE burden in ST2250 was comparable to ST59 but lower than ST8 and ST239 (mean 5.60 vs 6.70 for ST8; 5.60 vs 7.30 for ST239; *P* < 0.001; Fig. S6C). Relative to the epidemic *S. aureus* lineages, ST2250 lacked specific SaPIs, including SaPI2/SaPI3/SaPI4-like elements and SaPI5, the genomic island ACME, and the prophages φSa4 and φSa6, and it showed a lower prevalence of φSa3.

## DISCUSSION

Although *S. argenteus* was recognized as a distinct species around 2015 (4), it was not distinguished from *S. aureus*, and its global epidemiology remains incompletely understood. In our collection, *S. argenteus* accounted for 3.91% of presumptive *S. aureus* isolates, with the ST2250 lineage predominating (82.35%), a prevalence notably higher than that reported previously in East China (0.7%) (39) and Japan (0.55%) (40). Analysis of publicly available ST2250 genome data (*n* = 277, including 14 isolates from our collection) revealed that ST2250 represents a globally distributed lineage with ongoing transmission and potential environmental or community reservoirs and that its actual epidemiological burden has likely been underestimated due to historical non-differentiation from *S. aureus*.

Our phylogenomic analysis revealed that the global *S. argenteus* ST2250 population has diverged into three well-defined clades: clade I branched near the root around 1989, whereas clades II and III shared a recent common ancestor and diversified later, in about 1996 and 1997, respectively, reflecting a long evolutionary history and wide temporal spread. Each clade exhibited distinct geographical clustering, suggesting that ST2250 diversified through region-specific evolutionary events rather than a single global expansion. Phylogenomics together with paired SNP distance analysis revealed long-distance transmission, with clusters spanning Thailand, the United Kingdom, Vietnam, and Denmark, consistent with international spread via frequent human movement or other transmission vectors. Furthermore, non-human ST2250 genomes were phylogenetically intermixed with human-derived genomes, suggesting possible cross-host transmission and the presence of animal or food reservoirs (37). This aligns with a prior report of rapid dissemination of *S. argenteus* from food handlers to utensils (41) and with an epidemiological study of Chinese retail foods reporting a 7.2% isolation rate of *S. argenteus* (10), supporting the potential for foodborne or zoonotic transmission. Taken together, these findings point to an ecological network spanning hosts and continents that may facilitate the persistence and global spread of ST2250, underscoring the need for strengthened surveillance across countries and region-specific epidemiological control measures.

Interestingly, our origin analysis supports recent diversification of CC2250 from a deeply split ancestor, rather than recent formation by segmental recombination from CC75. Plausible origin scenarios include a host or ecological shift from an unsampled non-human or environmental reservoir into humans in the mid-1980s with subsequent expansion, or long-term low-level circulation with more recent ecological opportunity or mobile element gains facilitating spread and detection. Discriminating between these scenarios will require broader One Health sampling across animals, foods, environmental and wastewater sources, together with long-read sequencing to resolve structural variants and plasmidomes, metagenomic read mining for CC2250-like sequences, and longitudinal sampling in regions with high prevalence.

The methicillin-resistant phenotype of *S. argenteus* is relatively rare and exhibits regional variability (19). However, the evolutionary emergence and genomic features of MRSArg remain largely uncharacterized in a global context. Most ST2250 MRSArg in our data set belonged to clade II (94.06%), which formed a tightly clustered sublineage carrying SCC*mec* IVc, with *dfrG* (trimethoprim resistance) located within the element. These MRSArg genomes were closely related to MSSAarg genomes within the same clade, suggesting a single SCC*mec* acquisition event in the early 2000s, analogous to the evolution of other MRSA lineages (42, 43), where SCC*mec* was acquired once and the clone subsequently radiated.

Additionally, clade II MRSArg genomes universally carried a 20.6 kb multi-replicon plasmid pST2250-1, encoding *blaZ* and heavy metal resistance genes (*cadD*, *arsR*). Similarly, clade III MSSArg genomes formed a multidrug-resistant subclade that exclusively carried a related 30 kb plasmid pST2250-2, derived from pST2250-1, encoding *blaZ*, *tet*(L) (tetracycline resistance), *aph(3')-III* (aminoglycoside resistance), and the heavy metal resistance genes (*cadD*, *arsR,* and *czcD*). Both plasmids were highly similar to their *S. aureus* counterparts, implying interspecies plasmid transfer between *S. aureus* and *S. argenteus*. Importantly, the accumulation of antibiotic and heavy metal resistance determinants likely conferred a selective advantage under hospital-associated pressures, thereby facilitating the spread and persistence of clade II/III genomes in healthcare environments. Furthermore, clades II and III exhibited a higher prevalence of virulence factors compared to clade I, particularly the *esaG7* gene in the type VII secretion system, which encodes an antitoxin-like immunity protein that neutralizes the EsaD nuclease toxin, thereby maintaining T7SS stability and enhancing the competitive fitness and persistence of bacteria within host niches (44). In summary, the dominance of clades II and III in global ST2250 *S. argenteus* populations is likely driven by their expanded resistance and virulence gene profiles, thereby promoting their persistence and transmission.

Beyond SCC*mec* and plasmids, all ST2250 genomes harbored canonical genomic islands (vSaα/β/γ). Notably, we identified one clade II genome (H1864) that harbored φSPβ, introducing *sasX* (a critical determinant of MRSA pathogenic success [38]) into a previously unobserved genetic background. This observation underscores the potential for the co-circulating lineages of *S. argenteus* and *S. aureus* to exchange bacteriophages and transfer virulence determinants, potentially generating new epidemic variants adapted to competing populations. However, other large MGEs were relatively scarce. For instance, φSa3, which carries IEC genes such as *scn* and *sak*, was present in only 56.25%–77.33% of genomes across clades, much lower than its prevalence (>90%) in the *S. aureus* epidemic lineages (36). Furthermore, the SaPI2 was fragmented in over 95% of genomes, losing conserved regions responsible for regulation and replication. Considering the notably smaller accessory genome in *S. argenteus* compared to *S. aureus* (16), we hypothesize that active CRISPR-Cas immunity in ST2250 may limit the uptake of new MGEs. However, although type III-A CRISPR-Cas was highly prevalent in ST2250, we identified only 11 unique spacers within these CRISPR arrays. This low spacer diversity is consistent with shared ancestry of spacer arrays and limited recent spacer acquisition, rather than convergent acquisition across clades, and does not by itself indicate ongoing CRISPR activity. Together with prior work showing that *S. argenteus* genomes lacking CRISPR-Cas do not harbor more integrated prophages (17), these observations suggest that CRISPR is unlikely to be the sole driver of the MGE landscape in ST2250. We, therefore, interpret the reduced MGE burden as the combined effect of multiple defense systems, including type I RM and abortive infection, along with ecological and temporal factors. In particular, since the inferred origin of ST2250 dates to the mid 1980s, the lineage has had a shorter residence time in the human niche than *S. aureus*, which may also contribute to its smaller accessory pangenome. Functional assays and longitudinal sampling will be needed to quantify the relative contributions of these mechanisms.

Interestingly, pan-genome analysis revealed that ST2250 lineage has an open genome, with accessory genes continuing to accumulate over time. This pattern may reflect a balance under strong selective pressures: while ST2250 is capable of acquiring beneficial genes, such as plasmid-encoded resistance determinants, the defense systems may limit the integration of random MGEs. In addition, signals of convergent evolution support the idea that ST2250 is adapting to the hospital setting. We identified 39 genes under positive selection across the lineage, spanning functional categories of antibiotic resistance, bacterial virulence, and central metabolism. This distribution suggests that ST2250 faces multiple selective pressures from both antimicrobial exposure and host immune responses, potentially driving selection for traits that enhance both resistance and overall fitness. Similar multigenic adaptations have been observed in hospital-adapted strains of *S. aureus* and other pathogens subjected to high antibiotic pressure (45, 46). Although experimental validation is still needed, these positively selected mutations likely contribute to the persistence and pathogenicity of ST2250 in nosocomial environments.

This study has several limitations. First, sampling bias is inevitable: nearly half of the global genomes analyzed originate from the Netherlands and Thailand, potentially overrepresenting local diversity and underrepresenting other regions. In addition, public genomes may include copy or serial isolates that cannot be confidently identified across studies, further overrepresenting specific outbreaks or regions. Second, inferred transmission events are based solely on SNP thresholds and phylogenetic proximity, without supporting epidemiological data; thus, transmission directionality and person-to-person spread cannot be confirmed. Third, although the study includes a One Health perspective, non-human genomes are limited (13 out of 277) and represent a narrow range of hosts, which constrains conclusions about cross-species transmission. Future work should broaden geographic and host sampling (including animal and environmental sources) and incorporate functional studies to elucidate the evolutionary dynamics and adaptive mechanisms underlying the global success of ST2250. Additionally, our cross-lineage comparison with *S. aureus* is preliminary and limited to ST59, ST8, and ST239; a comprehensive and balanced cross-species, multi-lineage analysis that includes additional *S. argenteus* lineages and a broader set of *S. aureus* lineages is warranted, together with a formal reconstruction of the ~1986 ancestor, including ancestral core sequence and accessory gene content under explicit gain/loss and HGT models.

In conclusion, this study provides a comprehensive genomic analysis of the globally distributed *S. argenteus* ST2250 lineage, integrating local clinical isolates with international genome data. We demonstrate that ST2250 has diversified into a basal clade I and sister clades II and III with distinct geographic structures, reflecting region-specific evolutionary events and sustained global spread over the past three decades. Frequent cross-country, intercontinental, and cross-host transmission events were identified, highlighting the ecological versatility of this lineage. Methicillin resistance was almost exclusively confined to clade II, which harbored SCC*mec* IVc and a *blaZ*-encoding plasmid, while clade III formed a distinct MDR subclade enriched for other resistance determinants. Both clades II and III also showed increased virulence gene content, suggesting parallel adaptations to hospital-associated environments through different evolutionary strategies. Despite active defense systems that might limit the acquisition of MGEs, the ST2250 pan-genome remains open, with continued gene flux and convergent evolutionary signals targeting resistance and metabolism-related pathways. These findings collectively reveal a dynamic interplay between antimicrobial selection, genomic plasticity, and ecological adaptation underlying the global success of ST2250.

## Data Availability

The whole-genome sequences of 14 ST2250 *S. argenteus* isolates determined in this study were submitted to the DDBJ/EMBL/GenBank database under BioProject accession PRJNA1347620.

## SUPPLEMENTAL MATERIAL

Supplemental figures aac.01628-25-s0001.pdf Fig. S1 to S6.

Table S1 aac.01628-25-s0002.xlsx Information of 277 ST2250 genomes analyzed in this study.

Table S2 aac.01628-25-s0003.xlsx Homology analysis results of spacers from *S. argenteus* ST2250 genomes.

Table S3 aac.01628-25-s0004.xlsx Genes predicted to be under selection in the ST2250 population.

ASM does not own the copyrights to Supplemental Material that may be linked to, or accessed through, an article. The authors have granted ASM a non-exclusive, world-wide license to publish the Supplemental Material files. Please contact the corresponding author directly for reuse.
