# Supplementary material for: Emergence and genomic adaptation of the globally disseminated ST2250 lineage within the Staphylococcus aureus complex
Source: Antimicrob Agents Chemother. 2026 Jan 26;70(3):e01628-25. doi: 10.1128/aac.01628-25 (PMC12959139; doi:10.1128/aac.01628-25)
Supplement: Supplemental figures — Fig. S1 to S6. [file aac.01628-25-s0001.pdf]

Emergence and genomic adaptation of the globally disseminated ST2250 lineage within the *Staphylococcus aureus* complex

Supplementary Figures

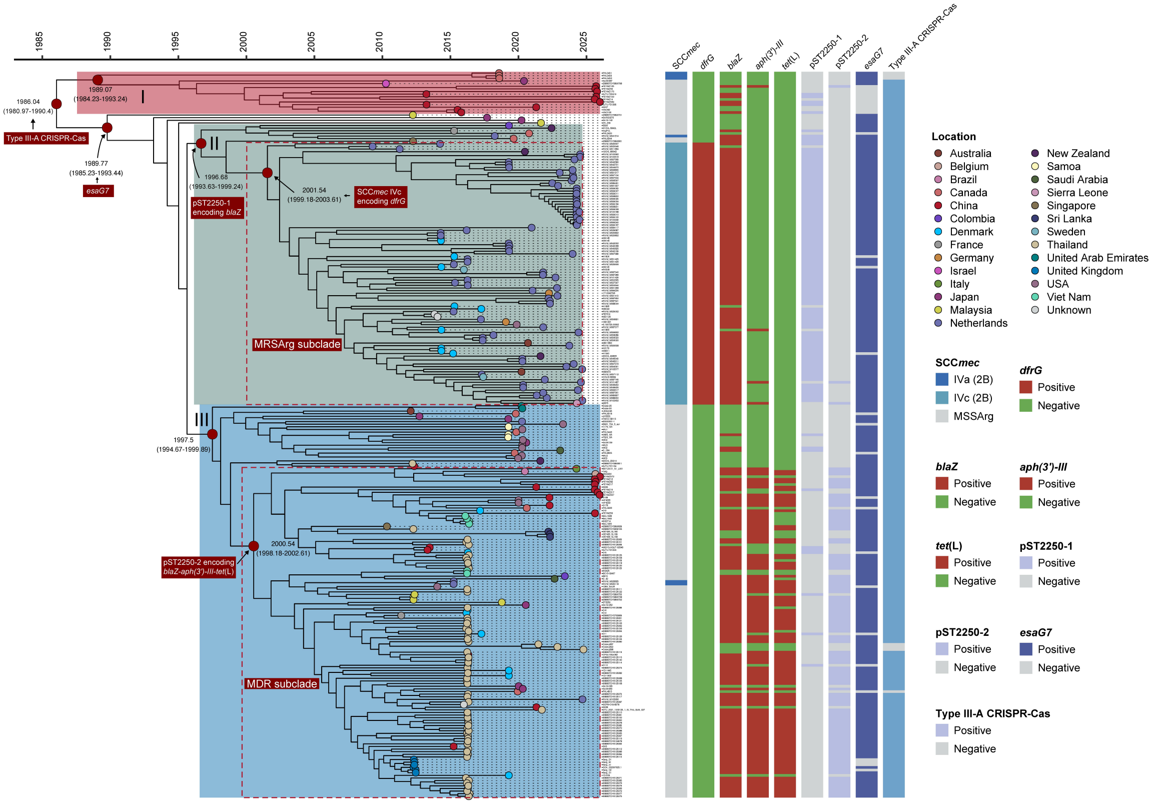

Figure S1. Dated phylogenetic reconstruction of ST2250 genomes. Tip nodes are colored by geographic origin. Clade I is basal, and clades II and III share a recent common ancestor. The three clades are shaded with different background colors. The characteristics of each genome are shown on the right, including SCC*mec* type, presence of *dfrG*, *blaZ*, *aph(3')-III*, *tet*(L), pST2250-1, pST2250-2, *esaG7*, and type III-A CRISPR-Cas. Main evolutionary events are exhibited in red boxes, with relevant divergence time and 95% HPD intervals shown at the nodes. The MRSArg subclade in clade II and the MDR subclade in clade III are outlined in dashed red boxes.

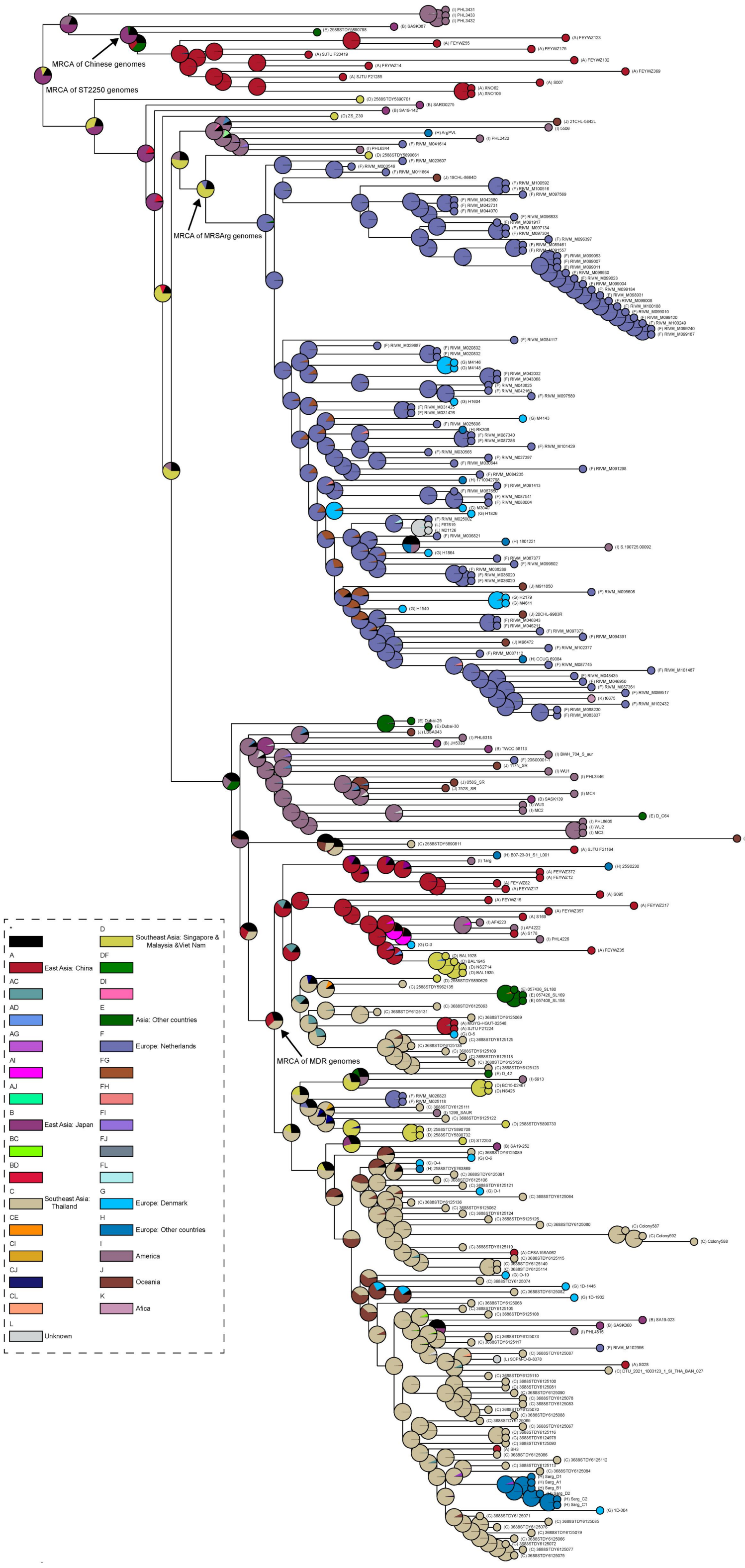

Figure S2. Phylogeographic analysis of global ST2250 isolates based on the Bayesian Binary MCMC method in RASP v4.2.

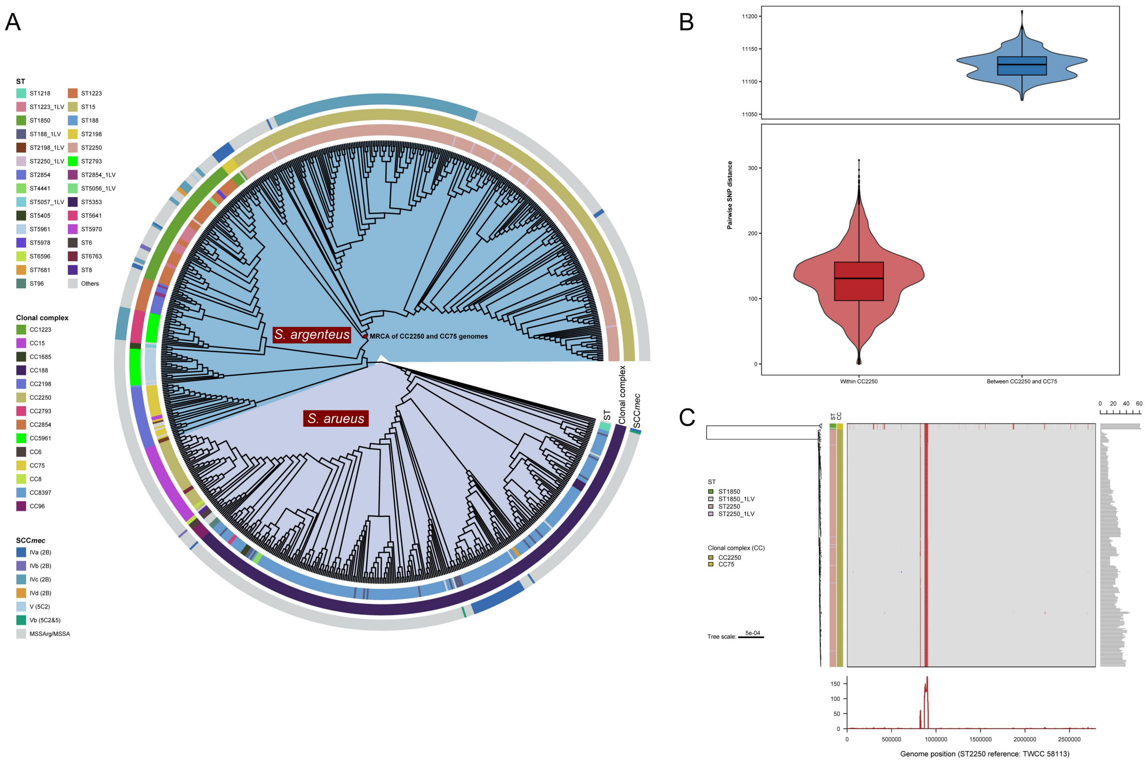

Figure S3. Phylogeny and recombination of CC2250 relative to public *Staphylococcus* genomes. (A) Core-genome phylogeny of the 500 Mash nearest genomes to TWCC 58113 (queried against 85,893 *S. aureus* and 173 non-ST2250 *S. argenteus* assemblies) together with the 277 ST2250 genomes analyzed in this study. The circular tree is annotated from the inside out with ST, clonal complex, and SCC*mec* type. (B) Pairwise core-SNP distances within CC2250 and between CC2250 and the closest external lineage CC75. (C) Recombination between CC2250 and CC75. The left panel presents a maximum-likelihood tree of CC2250 and CC75 genomes, while the adjacent heatmap depicts recombination density along the reference genome. The line plot below the recombination heatmap displays the frequency of recombination events across the reference genome. To the right of the heatmap, a barplot quantifies the total number of recombination events per genome.

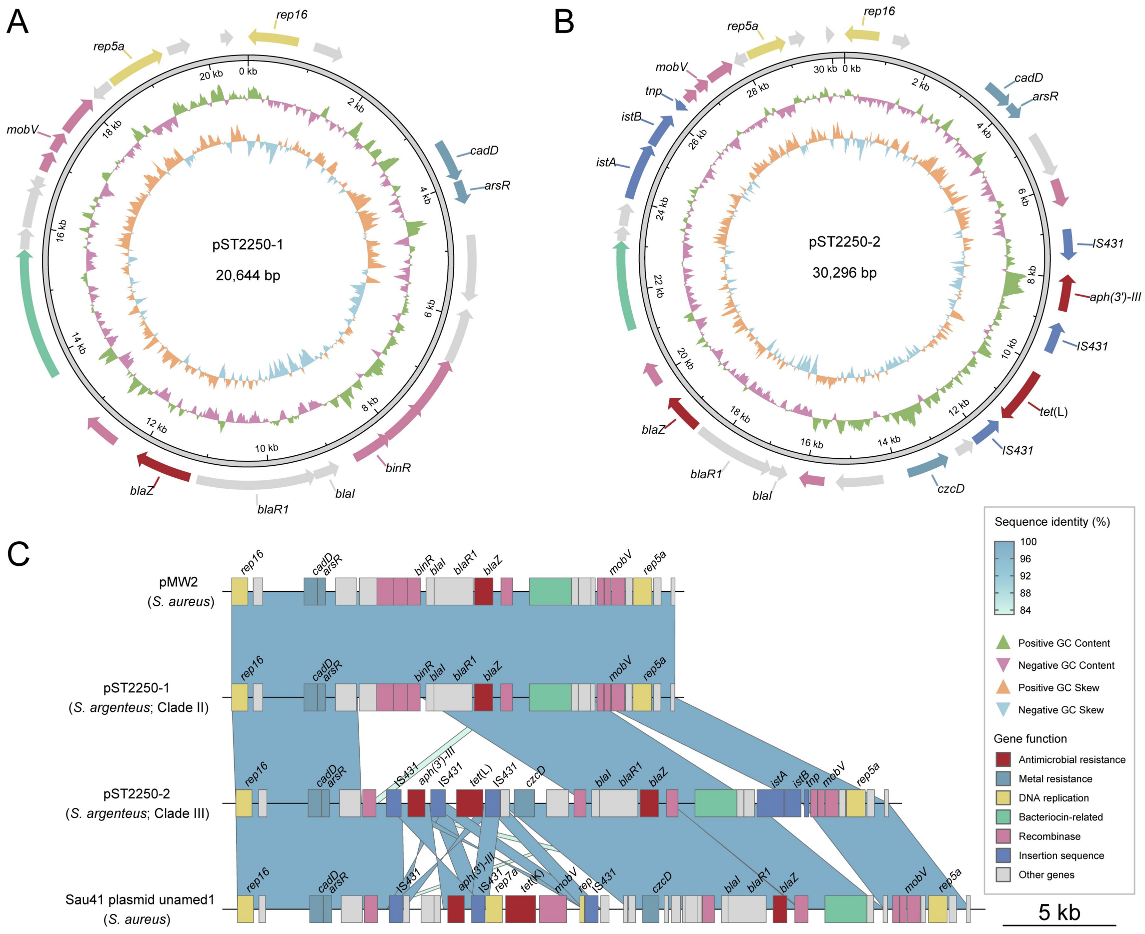

Figure S4. Structural analysis of ST2250-associated plasmids. (A-B) Circular maps of pST2250-1 and pST2250-2 plasmids, showing coding sequences, GC content, and GC skew. (C) Linear comparison of pST2250-1 and pST2250-2 with closely related plasmids from *S. aureus*. Genes are colored by functional category.

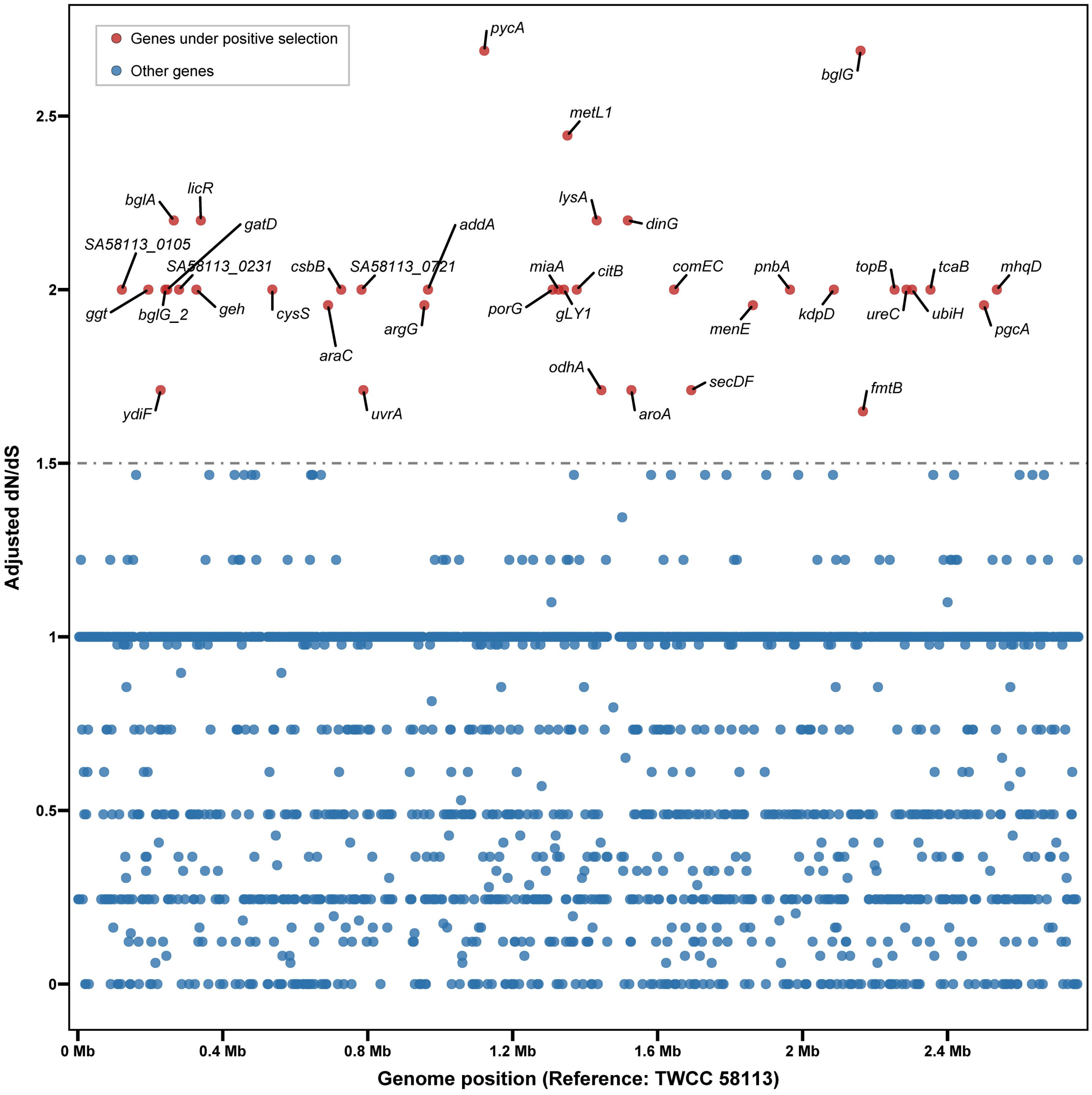

Figure S5. Genome-wide analysis of positive selection across the ST2250 lineage. Each dot represents a gene plotted by its chromosomal position and adjusted dN/dS ratio. Genes under positive selection are highlighted in red. To avoid infinite dN/dS values when no synonymous substitution was detected, genes with $S = 0$ were assigned a value of 2 if $N \geq 7$, and 1 otherwise.

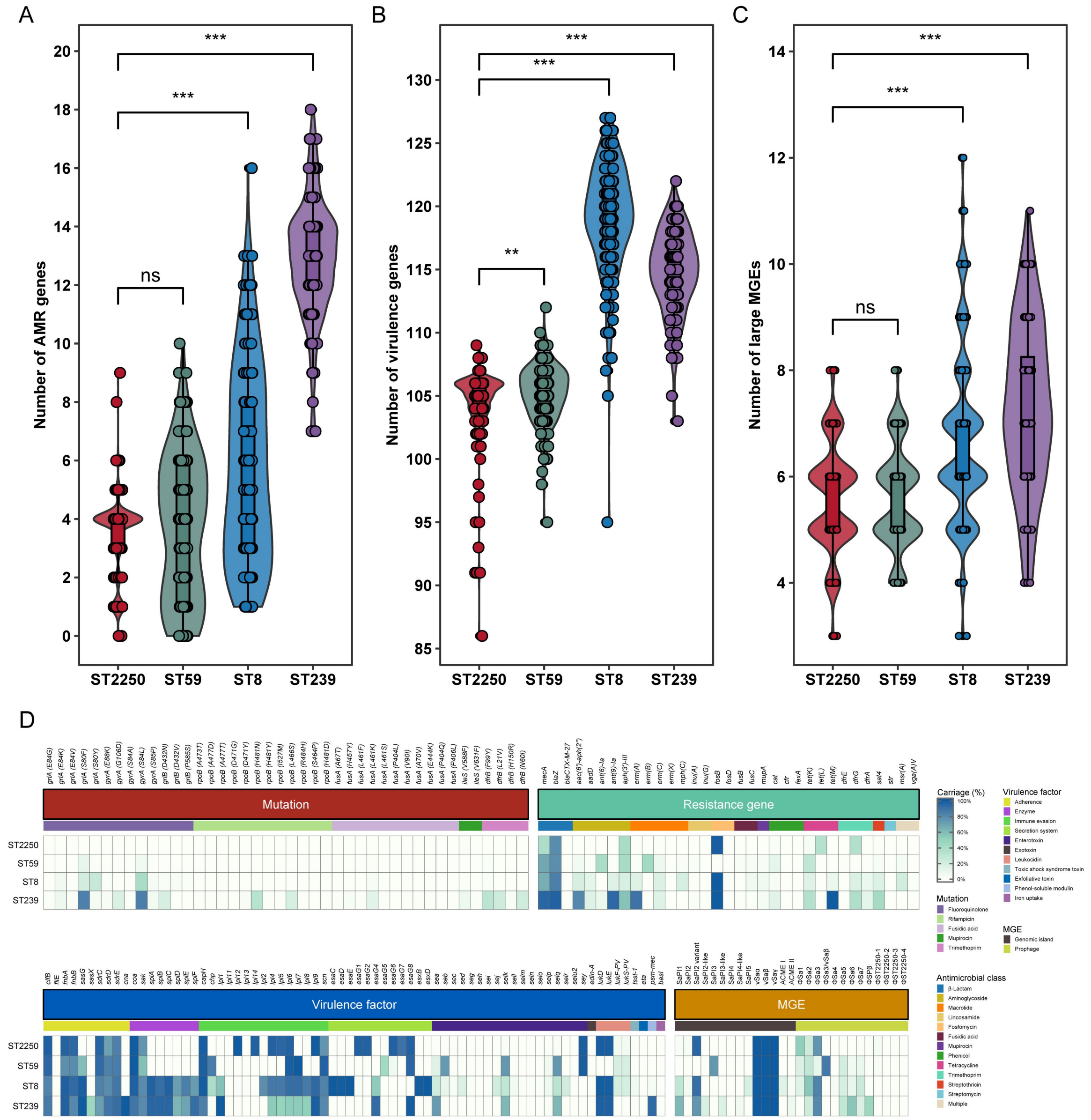

Figure S6. Comparison of ST2250 with *S. aureus* epidemic lineages ST59, ST8 and ST239 for AMR genes, virulence genes, and large MGEs. (A-C) Violin plots comparing the number of AMR genes (A), virulence factors (B), and large MGEs (C) between ST2250 and *S. aureus* lineages. **, $p < 0.01$; ***, $p < 0.001$. ns, not significant. (D) Heatmap illustrating the distribution of mutations, AMR genes, virulence factors and MGEs across ST2250 and *S. aureus* lineages. Cells are shaded by carriage frequency

(percentage of isolates carrying the trait) within each lineage. For clarity of presentation, virulence genes with a carriage frequency over 90% across all lineages are not shown in the figure.
